# Multi-Index Quantitative Analysis of Pharyngiyan Tablet Characteristics by Screening Different Quality Control Components

**DOI:** 10.1155/jamc/6691730

**Published:** 2025-04-25

**Authors:** Huan Cao, Liang Zhang, Xiaoning Zhan, Nan Hu, Xueyan Bi, Yanhong Wang

**Affiliations:** ^1^School of Pharmacy, Heilongjiang University of Chinese Medicine, Heilongjiang, Harbin 150040, China; ^2^NMPA Key Laboratory for Quality Research and Evaluation of Traditional Chinese Medicine, Heilongjiang Institute for Drug Control, Heilongjiang, Harbin 150088, China; ^3^Laboratory of Traditional Chinese Medicine, Heilongjiang Institute for Drug Control, Heilongjiang, Harbin 150088, China; ^4^Pharmacy Department, Harbin Traditional Chinese Medicine Hospital, Heilongjiang, Harbin 150010, China

**Keywords:** index screening, pharyngiyan tablets, quality evaluation, quantitative analysis, total composition analysis

## Abstract

Pharyngiyan tablet is a traditional Chinese medicine (TCM) renowned for its efficacy in moisturizing the lungs, clearing the throat. However, it exhibits quality variations due to discrepancies in regulatory standards and challenges in comprehensive evaluation. In this study, ultra-performance liquid chromatography–quadrupole time-of-flight tandem mass spectrometry (UPLC–Q–TOF–MS/MS) was used to qualitatively analyze the chemical composition of pharyngiyan tablets. Full composition analysis detected 131 chemical constituents. Gallic acid, paeoniflorin, rutin, baicalin, and harpagoside were identified as quality control (QC) indexes through rational screening. These indexes were selected based on their effectiveness in demonstrating differences, stability after processing, specificity, and cost-effectiveness in measurement. For the multimetric quality assessment of the five screening components, a high-performance liquid chromatography with diode array detector (HPLC–DAD) method with wavelength switching was developed. A total of 104 batches of samples from 10 manufacturers were included in the analyses. The content of each ingredient varied significantly across enterprises and batches, with certain enterprises characterized by inferior drug feeding, deviation from prescribed ingredient amounts, and nonstandardized feeding practices. Overall, aquality assessment method was established to provide a basis for the market regulation and quality assessment of pharyngiyan tablets.

## 1. Introduction

Traditional Chinese medicine (TCM) is pivotal in global traditional medicine owing to its holistic perspective, offering distinct advantages in treating complex chronic diseases [1–3]. Pharyngiyan tablets effectively nourish Yin and moisturize the lungs while clearing the throat, suppressing cough, and relieving itching. The formula of these tablets comprises 12 Chinese herbal medicines, including *Scrophularia ningpoensis Hemsl,* Moutan Cortex, and Canarii Fructus.

The existing benchmark is outlined in the guidelines established by the Ministry of Medicine, Book 2 [4], covering the description, identification, inspection criteria, and assay deficiencies. A comparison of enterprise registration standards [5–11] indicates discrepancies in quantitative indexes, limits, and testing methods for similar herbs. Therefore, conducting a comprehensive evaluation of pharyngiyan tablet quality across enterprises remains challenging. Notably, to determine the pharyngitis tablet contents, the researchers selected different indicators [12, 13] without providing a sufficiently detailed and clear explanation for the selection criteria of choosing the given indicators.

The essence of Chinese medicine regulatory science relies on integrating traditional attributes with innovative research methods, tools, and standards through cross-border integration. Establishing quality control (QC) standards using quality markers for identification has gained industry recognition. QC, crucial for internal enterprise management, requires selecting indicators for drug quality assessment beyond the mere identification of quality marker components. It necessitates active ingredients with traceable quantitative values from raw material drugs to finished products, stability in the composition of preparation processing technology, reasonable detection costs, and addressing other elements. These measurements collectively aim to achieve comprehensive QC across the entire industrial chain [14–16].

In selecting a QC index for pharyngiyan tablets, integrating comprehensive component analysis and research findings on active ingredients is essential. Pharyngiyan tablets primarily contain flavonoids, terpenes and their glycosides, and organic acids [17], known for their anti-inflammatory and analgesic efficacy in treating pharyngitis. The TCM effector mechanism results from the collective effect of various components. Screening the three main quality-differentiating compounds aims at identifying key quality attributes within the overall composition, aligning with the TCM characteristics that involve multiple components, targets, and medicinal efficacy attributes [18]. Compounds such as baicalin, the active ingredient in rutin, and other flavonoids can effectively alleviate pathogenic infections, reducing heat, detoxifying toxins, inhibiting bacteria, and providing anti-inflammatory effects [19–21]. Polyphenols, such as gallic and ellagic acids, are suggestibly the key to “clearing heat and improving the throat” [22, 23]. Terpenoids, such as harpagoside, exert inhibitory effects on inflammatory factor release in human bronchial epithelial cells [24, 25]. Screening compounds with a stable production process is crucial for identifying measurable and controllable substances throughout extraction and processing at each stage. Screening low-cost measurable compounds involves utilizing controlled substances from the National Institutes for Food and Drug Control (NIFDC), known for their high purity, to reduce testing costs.

In the present study, we aimed to establish a high-performance liquid chromatography with diode array detector (HPLC–DAD) wavelength–switching multi-indicator method to assess the quality of pharyngiyan tablets by screening indicator substances. Consequently, we achieved the cost-effective, efficient, comprehensive, and scientifically sound QC of pharyngiyan tablets.

## 2. Materials and Methods

### 2.1. Materials, Chemicals, and Reagents

Overall, 104 sample batches from 10 manufacturers were sourced from the national sampling and testing link. The manufacturers were denoted by the letters A–J, with 15 batches from manufacturer A, 6 from B, 10 from C, 23 from D, 5 from enterprise E, 11 from F, 7 from G, 15 from H, 4 from I, and 8 from J.

Gallic acid (batch no. 110831–201906, purity: 91.5%), paeoniflorin (batch no. 110736–202145, purity: 94.6%), rutin (batch no. 100080–202012, purity: 91.6%), baicalin (batch no. 110715–202122, purity: 94.2%), and harpagoside (batch no. 111730–202110, purity: 96.8%) were procured from the NIFDC. We used methanol, acetonitrile, and formic acid of mass spectrometry grade, chromatographic-grade phosphoric acid, and distilled water (Watsons).

### 2.2. Compositional Analysis via Ultra-Performance Liquid Chromatography–Quadrupole Time-of-Flight Tandem Mass Spectrometry (UPLC–Q–TOF–MS/MS)

#### 2.2.1. Preparation of the Test Solution

Ten pharyngiyan tablets were taken, and their coatings were removed; the tablets were then ground, and approximately 2.0 g of the powder was transferred to a stoppered conical flask. Subsequently, 25 mL of methanol was added, followed by ultrasonic treatment for 30 min. The mixture was then cooled and centrifuged at 12,000 rpm for 10 min. The supernatant was collected, obtaining the desired extract.

#### 2.2.2. Preparation of the QC Sample Solution

One sample was taken from each of the 10 enterprises, i.e., 10 samples according to the method described in Subsection “2.2.1.” to prepare the test solutions. Next, 1 mL was taken from each of the 10 samples and mixed well.

#### 2.2.3. UPLC Conditions

The instruments used in this study included the ACQUITY™ UPLC (Waters Corporation, United States of America). The samples were subjected to separation on an ACQUITY™ UPLC BEH C_18_ column (2.1 × 50 mm, 1.7 μm) at 35°C. The mobile phase consisted of 0.1% formic acid aqueous solution (A) and acetonitrile (B), utilizing a gradient elution (0–15 min, 95%–5% A) at a flow rate of 0.4 mL min^−1^. Importantly, reversed-phase chromatographic systems commonly use acetonitrile–water systems [26]. Formic acid was added to the samples to increase their ionization. The temperature of the experimental room was maintained at 4°C, and the injection volume was 4 μL.

#### 2.2.4. Mass Spectrometry Conditions

**Figure 1:**
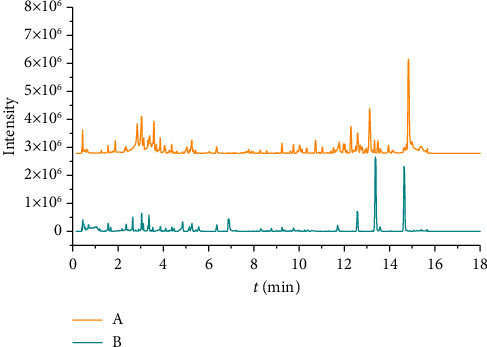
BPI chromatograms of QC sample in (A) positive and (B) negative ion modes.

MSE data were collected using an electrospray ionization (ESI) for both positive and negative ions. The ion source temperature was maintained at 110°C with a cone-pore sampling voltage of 40 V. In the positive ion mode, the capillary voltage was set at 3.0 kV, desolvation at 650 L/h, and desolvation temperature at 350°C, with a cone hole gas flow rate of 50 L/h. In the negative ion mode, the capillary voltage was set at 2.5 kV, desolvation at 800 L/h, desolvation temperature at 450°C, and the cone-pore gas flow at 100 L/h. The scanning mode applied was a full scan, with a mass data acquisition range of m/z 50–1200, utilizing the centroid acquisition mode.  Figure 1 illustrates the QC chromatogram under the specified chromatographic and mass spectrometric conditions.

#### 2.2.5. Component Analysis and Attribution

Based on the method described, the chromatogram of the pharyngiyan tablets' QC sample was imported into the UNIFI analysis platform. Subsequently, automated data processing and database searching were conducted. The analysis revealed multiple candidates matching the quasimolecular ions at low collision energies with an accurate mass error of < 5 mDa. Further analysis involved exploring the structure of each secondary fragment corresponding to the parent ions at high collision energies in the diagram to determine accuracy. If the cleavage appeared reasonable, the structure could be initially concluded to be correct, thus confirming the compound. Identification of the composition relied on UNIFI autoidentification, literature reports, and comparison with online and offline mass spectrometry databases.

### 2.3. Establishment of the Multi-Indicator Content Determination Method

In this study, we investigated conditions including wavelength, elution gradient, and extraction method of the test solution. The wavelength variation range was determined based on the ultraviolet (UV) absorption maximum of the target components. Subsequently, the optimal wavelength for each phase was determined, considering factors such as peak response value, shape, and impurity interference. Gradient changes in the mobile phase were examined using an acetonitrile–0.1% phosphoric acid aqueous solution over time. Conditions were selected to achieve good peak shape and high separation, leading to the choice of gradient elution with acetonitrile–0.1% phosphoric acid aqueous solution as the mobile phase. We also assessed factors such as the test solution extraction solvent, method (ultrasonic and reflux), and time. Ultimately, the decision was made to use methanol as the extraction agent and to conduct ultrasonic extraction for 60 min.

#### 2.3.1. Reference Solution

Gallic acid, paeoniflorin, rutin, baicalin, and harpagoside were weighed at 102.60, 20.78, 20.18, 10.08, and 10.46 mg; dissolved in methanol; and fixed in a 100-mL volumetric flask to prepare the reference solutions. To obtain the final concentration, the reference solution was diluted 10-fold.

#### 2.3.2. Test Solution

Twenty pharyngiyan tablets were taken, and their coatings were removed. Subsequently, we precisely weighed and finely ground the tablets into a powder. Approximately 2.0 g of the powder was precisely weighed and placed into a stoppered conical flask. Thereafter, 25 mL of methanol was added, and the flask was tightly closed and weighed. The mixture underwent ultrasonic treatment (power: 250 W and frequency: 35 kHz) for 60 min, followed by cooling and reweighing. Methanol was added to compensate for any weight loss, and the mixture was shaken before filtration. Finally, the filtrate was obtained, constituting the desired extract.

#### 2.3.3. Control Solution

We prepared the negative samples except for Canarii Fructus, Moutan Cortex, Farfarae Flos, Oroxyli Semen, and Scrophulariae Radix following the prescription process for each and prepared a control solution following the procedure outlined in Section 2.3.2.

#### 2.3.4. Chromatographic Conditions and System Adaptability

The chromatographic conditions applied an Agilent 1100 HPLC (Agilent) with a Kromasil-100-5-C_18_ (4.6 mm × 250 mm, 5 μm) column. The mobile phase consisted of acetonitrile (A) and 0.1% aqueous phosphoric acid (B), applying gradient elution (0–10 min, 3% A; 10–11 min, 3% A ⟶ 15% A; 11–20 min, 15% A ⟶ 20% A; 20–50 min, 20% A ⟶ 60% A; 50 min, 20% A ⟶ 60% A). Detection wavelengths were set at 210 nm for 0–6 min, 272 nm for gallic acid for 6–13 min, 245 nm for 13–18 min, 230 nm for paeoniflorin for 18–22.5 min, 320 nm for rutin for 22.5–28 min, and 278 nm for baicalin and harpagoside for 28–50 min. The flow rate was maintained at 1.0 mL/min with a column temperature of 30°C. The injection volume was 5 μL.

### 2.4. Method Validation

#### 2.4.1. Linear Relationship

A series of concentrations of the mixed solutions were prepared by dissolving the accurately weighed and appropriate amounts of each reference in methanol. The mixed solutions contained gallic acid (4.14, 8.28, 16.56, 33.12, and 165.62 μg/mL), paeoniflorin (5.39, 10.78, 21.57, 43.14, and 215.69 μg/mL), rutin (5.47, 10.95, 21.89, 43.78, and 218.92 μg/mL), baicalin (7.04, 14.08, 28.17, 56.33, and 281.66 μg/mL), and harpagoside (5.35, 10.70, 21.39, 42.79, 213.93 μg/mL). A sample volume of 5 μL was taken for the measurements and unitary linear regression was used for the analysis with correlation coefficient values above 0.9990. The results met the requirements of the standard drug quality analysis method of the Chinese Pharmacopoeia.  Table 1 presents the results of the linear equation.

#### 2.4.2. Precision Test

Appropriate quantities of gallic acid, paeoniflorin, rutin, baicalin, and harpagoside control were prepared, yielding concentrations of 100.65, 19.658, 18.485, 9.4954, and 10.125 μg/mL, respectively, and consecutively injected into the sample six times following the method outlined in Section 2.3.4. The results indicated good instrument precision.

#### 2.4.3. Repeatability Test

We prepared six samples individually from the same batch of pharyngiyan tablets following the method outlined in Section 2.3.2. The chromatographic conditions specified in Section 2.3.4 were applied to determine the average contents of gallic acid, paeoniflorin, rutin, baicalin, and harpagoside, which were 1.738, 1.063, 0.1077, 0.2487, and 0.1561 mg/g, respectively, with RSDs ≤ 2.0%, indicating good reproducibility of the method.

#### 2.4.4. Stability Test

The pharyngiyan tablet samples from the same batch were utilized to prepare the test solution following the procedure outlined in Section 2.3.2. Chromatographic conditions specified in Section 2.3.4 were applied to determine the peak areas at 0, 3, 12, 23, 30, and 37 h. The results demonstrated that the stability of the test solution remained satisfactory within 37 h.

#### 2.4.5. Recovery Rate Test

We collected approximately 1 g of the pharyngiyan tablets powder with a known amount of the five ingredients. In total, six portions were collected. A known quantity of reference solution was added following the parallel preparation method of the test solution outlined in Section 2.3.2 and according to the chromatographic conditions for determination described in Section 2.3.4. Reference substances with known purity were used for the determination of the recovery of the added sample. The recovery ratio was calculated by the margin of the determined value and the amount of the examined substance divided by the amount of the added reference substance.  Table 2 presents the results, including the calculated recovery.

#### 2.4.6. Specificity Test

**Figure 2:**
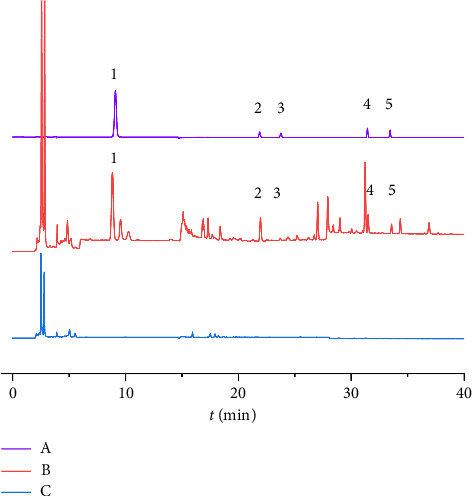
Chromatogram of determination of pharyngiyan tablet contents. (A) Reference solution. (B) Test solution. (C) Control solution. 1. Gallic acid. 2. Paeoniflorin. 3. Rutin. 4. Baicalin. 5. Harpagoside.

**Figure 3:**
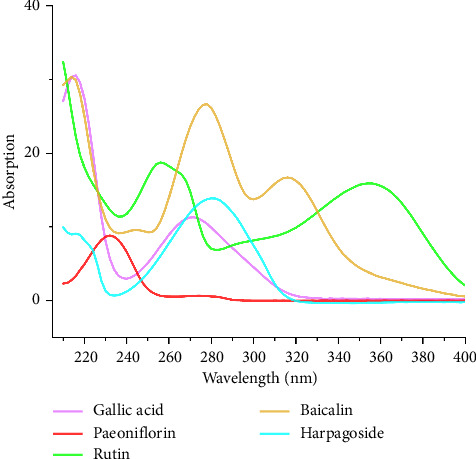
UV full-wavelength scanning spectra of multi-indicator components.

The reference, test, and control solutions were prepared according to the chromatographic conditions outlined in Section 2.3.4. A chromatogram and absorption spectra were recorded for each solution.  Figure 2 displays the chromatogram of each solution, and Figure 3 presents the spectral results of the control, demonstrating the absence of interference from the control solution.

#### 2.4.7. Sample Analysis

A total of 104 batches of pharyngiyan tablet samples were individually prepared into test solutions following the method outlined in Section 2.3.2. The samples were determined under the conditions specified in Section 2.3.4, applying the external standard method to calculate the content of each tablet. The content was calculated based on the number of indicator components present in each tablet.

## 3. Results and Discussion

### 3.1. UPLC–TOF–MS/MS Compositional Analysis Results

UPLC–Q–TOF–MS/MS was applied to analyze samples from each enterprise, detecting 131 chemical components in the QC samples. These components included 31 flavonoids, 28 terpenoids and their glycosides, 13 organic acids, 11 volatile oils, 8 alkaloids, 8 saponins, 5 glycosides, 3 acetophenones, 3 amino acids, 3 tannins, 3 triterpenoids, 3 fatty acids, 3 saccharides, 2 vitamins, 2 quinones, 1 furan, 1 indole, 1 organic ester, 1 phenylpropanoid, and 1 stilbene.  Table 3 summarizes the results.

### 3.2. Selection of QC Indicator Components for Pharyngiyan Tablets

The screening process involved selecting 31 flavonoids, 28 terpenes and their glycosides, and 13 organic acids from the entire component analysis of QC samples. The results of these three component types from samples of each enterprise were analyzed, considering variations in process parameters. Common components stable and measurable across different enterprises were identified based on their response intensity. Subsequently, the three common component types (10 flavonoids, 11 terpenes, their glycosides, and 6 organic acids) were analyzed using orthogonal partial least squares discriminant analysis (OPLS–DA) data. Components with a variable importance in projection (VIP) value > 1 were selected as the primary components to differentiate the quality difference between enterprises. Ingredients were screened for simultaneous fulfillment of the following conditions: attributability, uniqueness, low production process impact, high measurability, and low control costs. Ultimately, five index components, gallic acid, paeoniflorin, rutin, baicalin, and harpagoside, were selected based on the conditions above.

### 3.3. Analysis of the Multi-Indicator Content Determination Results

**Figure 4:**
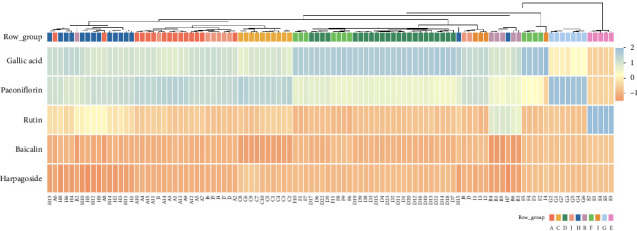
Results of HCA for multi-indicator content determination.

**Figure 5:**
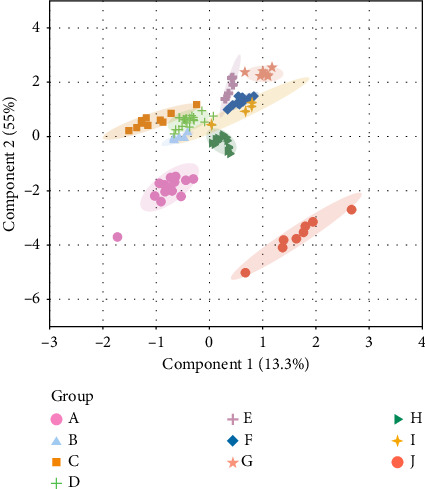
Graph of OPLS–DA results of multi-indicator content determination.

**Figure 6:**
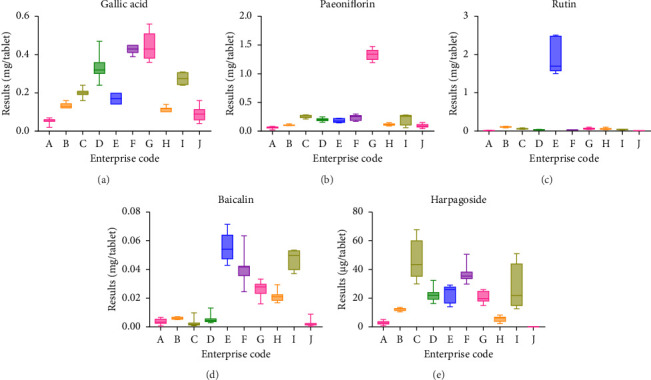
Box plot of the results of multi-indicator content determination. (a) Gallic Acid. (b) Paeoniflorin. (c) Rutin. (d) Baicalin. (e) Harpagoside.

**Figure 7:**
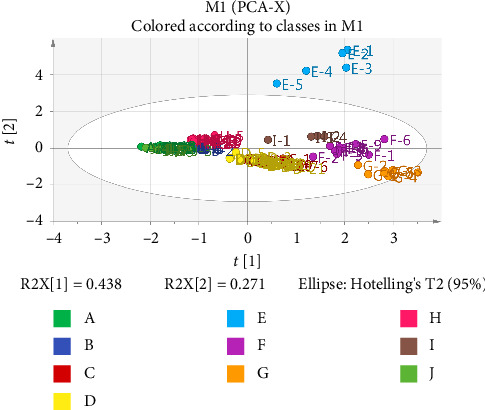
PCA score graph of the results of multi-indicator content determination.

The hierarchical cluster analysis (HCA) results showed that the samples used were divided into two categories, one corresponding to enterprise E and the other for the remaining enterprises (Figure 4). The OPLS–DA results showed well-defined clustering of the firms, forming elongated oval shapes (Figure 5). This finding suggested that the differences between groups were greater than the variation between batches in groups. Results are represented using a boxplot generated with GraphPad (Figure 6). The content of the same ingredient varied significantly across enterprises. Specifically, the quantity of paeoniflorin was higher in Enterprise G, while the amount of rutin was unusually high in Enterprise E. The multi-indicator data were subjected to principal component analysis (PCA) using SIMCA software.  Figure 7 shows the results. The increased and more distant dispersion observed between different color groups showed more significant quality disparities among samples from various manufacturers. The scores of each batch of Enterprise E were clear outliers, warranting a focus on the quality of its products. Variations among results from batches of the same enterprise were minimal, demonstrating minimal differences between batches and a stable enterprise process.

From the results of multi-indicator content determination, the variations in content indicators among samples from different manufacturers were significant (Figure 6). For instance, the rutin components in E enterprises were nearly 100 times higher than those in other manufacturers. In addition, the total flavonoids in the samples of the enterprise originated from the rutin component rather than the cumulative total of its flavonoid components, as demonstrated by the results of previous experiments of the group. This result indicated a potential risk of Enterprise E illegally adding rutin monomers to pass standard tests. The harpagoside content of Enterprise J was 0 μg, but comprehensive ingredient analysis detected the characteristic components of *Scrophulariae Radix*, confirming its presence in the product. Other values of Enterprise J were generally lower than those of other enterprises, and its effective components were thus potentially insufficient. This result could stem from inferior-quality raw materials, deviation from the prescribed amount of ingredients, or excessive destruction of active ingredients due to high-temperature steam concentration processes. Variations among enterprises may also arise from differences in extraction methods, equipment, crushing techniques, and other conditions. Therefore, relevant departments should urge manufacturers to control the quality of raw materials, conduct rigorous inspections, and adhere to standardized feeding practices.

Different from previous assays, in this study, we achieved high expression of different compound classes using whole-component analysis, rational selection of QC indicators, and a combination of wavelength-switching technology. In the case of the constituent (harpagoside) of the monarch drug *Scrophularia ningpoensis Hemsl*, for example, a comparison of the measurement results with previous data indicated a similar range of results, despite certain differences in the values, presumably caused by differences in the measurement method and the sample sources [12, 13].

## 4. Conclusions

In this study, we screened the QC indexes of pharyngitis tablets based on the concept of the holistic TCM view [27, 28] and the results of a whole-component analysis of Chinese patent medicine. We established an HPLC–DAD wavelength–switching method for the online determination of multi-indicator and multicategory components. When comparing the results of this study with those of previous publications [12, 13], it is important to point out that the present study overcomes the limitation of previous single-wavelength differences in the absorption degree of different substances and responds to the differences in quality of proprietary Chinese medicines by enlarging the sample size. This method allows for the rapid, economical, efficient, and comprehensive control and product quality evaluation of pharyngitis tablets at each enterprise and provides technical support for scientific research. However, unfortunately, ways to achieve consistency in Chinese patent medicine quality through this route should be urgently addressed.

## Figures and Tables

**Table 1:** Linear equation results.

Component	Linear range (μg)	Regression equation	*r*	IDL (ng)	MDL (μg/g)
Gallic acid	0.0207–0.8281	*y* = 4493.9*x* − 37.882	0.9999	1.28	3.19
Paeoniflorin	0.0270–1.0784	*y* = 1730.8*x* − 15.206	0.9999	1.99	4.98
Rutin	0.0274–1.0946	*y* = 1451.3*x* − 15.262	0.9999	2.35	5.86
Baicalin	0.0352–1.4083	*y* = 4645.6*x* − 118.130	0.9993	0.57	1.42
Harpagoside	0.0267–1.0696	*y* = 3255.9*x* − 31.491	0.9999	0.76	1.90

Abbreviations: IDL, instrument detection limit; MDL, method detection limit.

**Table 2:** Results of precision, stability, repeatability, and recovery tests (*n* = 6).

Components	Precision RSD (%)	Stability RSD (%)	Repeatability RSD (%)	Recovery	
				Mean (%)	RSD (%)
Gallic acid	1.2	1.3	1.8	97.64	1.1
Paeoniflorin	1.4	1.5	1.3	101.13	1.6
Rutin	1.2	1.3	2.0	98.26	1.4
Baicalin	1.1	1.2	1.7	86.65	2.1
Harpagoside	1.5	1.6	1.3	102.55	1.1

**Table 3:** Identification of the chemical constituents of pharyngiyan tablets in positive and negative ion modes.

Serial no.	Component name	Observed RT (min)	Categorization	Neutral mass (Da)	Mass error (mDa)	Adducts	Formula	Item tags	ESI modes	VIP value (peak number within a taxonomic group)
1	Ophiopogonone B	0.9	Flavonoids	312.09977	−3.1	+NH4	C_18_H_16_O_5_	Ophiopogonis Radix	Positive	
2	5,7-Dihydroxy-6-methyl-3-(4′-hydroxybenzyl) chroman-4-one	1.28	Flavonoids	300.09977	−0.3	+NH4	C_17_H_16_O_5_	Ophiopogonis Radix	Positive	0.159579 (C3)^∗^
3	5-Hydroxy-6,7,3′,4′-pentamethoxyflavone	1.32	Flavonoids	358.10525	3	+NH4	C_19_H_18_O_7_	Peppermint oil	Positive	
4	Ophiopogonone A	1.39	Flavonoids	326.07904	−0.2	+NH4	C_18_H_14_O_6_	Ophiopogonis Radix	Positive	
5	Kaempferol-3-O-β-D-glucopyranoside	2.56	Flavonoids	448.10056	−0.6	+H	C_10_H_10_O_4_	Moutan Cortex, Farfarae Flos	Positive	0.35588 (C7)^∗^
6	Rutin	2.56	Flavonoids	610.15338	0	+H, +Na	C_27_H_30_O_16_	Farfarae Flos	Positive	1.87403 (C9)^∗^
7	5,6,4′-Trihydroxy-7,8-dimethoxyflavone	2.82	Flavonoids	330.07395	−1.9	+H	C_17_H_14_O_7_	Peppermint oil	Positive	
8	Scutellarein	2.9	Flavonoids	286.04774	1	+H	C_15_H_10_O_6_	Oroxyli Semen	Positive	
9	Kaempferol	3.02	Flavonoids	286.04774	0.3	+H	C_15_H_10_O_6_	Farfarae Flos	Positive	0.21833 (C1)^∗^
10	Luteolin-7-O-β-D-glucopyranoside	3.15	Flavonoids	448.10056	0.5	+H	C_21_H_20_O_11_	Peppermint oil	Positive	
11	Chrysin-7-O-β-D-glucopyranoside	3.17	Flavonoids	416.11073	−0.2	+H	C_21_H_20_O_9_	Oroxyli Semen	Positive	0.239079 (C5)^∗^
12	Oroxin B	3.23	Flavonoids	594.15847	5	+H	C_27_H_30_O_15_	Oroxyli Semen	Positive	1.58092 (C8)^∗^
13	d-Catechin	3.44	Flavonoids	290.07904	−1.2	+H	C_15_H_14_O_6_	Moutan Cortex	Positive	0.139559 (C2)^∗^
14	Baicalin	3.75	Flavonoids	446.08491	−0.2	+H, +Na	C_21_H_18_O_11_	Oroxyli Semen	Positive	1.45997 (C6)^∗^
15	Hispidulin	3.95	Flavonoids	300.06339	−1.4	+H	C_16_H_12_O_6_	Oroxyli Semen	Positive	
16	Quercetin	4.02	Flavonoids	302.04265	−1.8	+NH4	C_15_H_10_O_7_	Canarii Fructus, Farfarae Flos	Positive	1.24181 (C4)^∗^
17	Acacetin	4.4	Flavonoids	284.06847	−2.4	+Na	C_16_H_12_O_5_	Peppermint oil	Positive	
18	Wogonoside	4.52	Flavonoids	460.10056	0.3	+H	C_22_H_20_O_11_	Oroxyli Semen	Positive	
19	Chrysin	6.29	Flavonoids	254.05791	−0.9	+H	C_15_H_10_O_4_	Oroxyli Semen	Positive	
20	Linarin (Buddleoside)	6.38	Flavonoids	592.17921	4.6	+Na	C_28_H_32_O_14_	Isatidis Radix	Positive	
21	5,6,7-Trihydroxy-3-(4′-hydroxybenzyl) chromone	13.32	Flavonoids	300.06339	2.6	+H	C_16_H_12_O_6_	Ophiopogonis Radix	Positive	
22	6-Aldehydo-isoophiopogonone B	0.49	Flavonoids	339.0907	3.3	-H	C_19_H_16_O_6_	Ophiopogonis Radix	Negative	
23	Methyl ophiopogonanone A	2.32	Flavonoids	341.1055	2.5	-H	C_19_H_18_O_6_	Ophiopogonis Radix	Negative	
24	5,4′-Dihydroxy-6,7,8-trimethoxyflavone	2.76	Flavonoids	343.0837	1.3	-H	C_18_H_16_O_7_	Peppermint oil	Negative	
25	Amentoflavone	2.94	Flavonoids	583.0868	−1.4	+HCOO	C_30_H_18_O_10_	Canarii Fructus	Negative	
26	5-*o*-Desmethylnobiletin	3.47	Flavonoids	387.1104	1.8	-H	C_20_H_20_O_8_	Peppermint oil	Negative	
27	Apigenol	4.9	Flavonoids	269.0463	0.8	-H	C_15_H_10_O_5_	Oroxyli Semen	Negative	
28	Diosmetin	5.26	Flavonoids	299.0538	−2.4	-H	C_16_H_12_O_6_	Peppermint oil	Negative	
29	6-Formyl-isoophiopogonanone A	5.33	Flavonoids	401.0867	−1.2	+HCOO	C_19_H_16_O_7_	Ophiopogonis Radix	Negative	
30	Acacetin-7-O-β-D-glucoside	5.38	Flavonoids	491.1218	2.3	+HCOO	C_22_H_22_O_10_	Peppermint oil	Negative	0.196651 (C10)
31	5-Hydroxy-7,8-dimethoxy-6-methyl-3-(3′,4′-dihydroxybenzyl) chroman-4-one	6.84	Flavonoids	359.1137	0	-H	C_19_H_20_O_7_	Ophiopogonis Radix	Negative	
32	Paeonisuffrone	0.38	Terpenoids	198.08921	−1.5	+NH4	C_10_H_14_O_4_	Moutan Cortex	Positive	0.287216 (F2)^∗^
33	Ningposide A	0.73	Terpenoids	382.12638	0.6	+NH4	C_18_H_22_O_9_	Scrophulariae Radix	Positive	
34	Paeoniflorigenone	0.89	Terpenoids	318.11034	0.8	+NH4	C_17_H_18_O_6_	Moutan Cortex	Positive	
35	Ningpogoside B	1.83	Terpenoids	332.14712	−2.1	+NH4	C_15_H_24_O_8_	Scrophulariae Radix	Positive	0.230129 (F4)^∗^
36	Mudanpioside I	2.08	Terpenoids	480.16316	4.5	+H, +Na	C_23_H_28_O_11_	Moutan Cortex	Positive	
37	Paeoniflorin	2.27	Terpenoids	480.16316	−4.1	+Na, +NH4	C_23_H_28_O_11_	Moutan Cortex	Positive	1.15349 (F5)^∗^
38	Mudanpioside E	2.66	Terpenoids	526.16864	4.2	+Na	C_24_H_30_O_13_	Moutan Cortex	Positive	0.515214 (F8)^∗^
39	14-Acetoxy-7β-senecioyloxy-notonipetranone	2.92	Terpenoids	376.22497	−2.7	+NH4	C_22_H_32_O_5_	Farfarae Flos	Positive	
40	Neotussilagolactone	3.58	Terpenoids	344.19876	−0.6	+NH4	C_21_H_28_O_4_	Farfarae Flos	Positive	
41	Benzoyl-oxypaeoniflorin	3.76	Terpenoids	600.18429	−4.1	+Na	C_30_H_32_O_13_	Moutan Cortex	Positive	0.387792 (F11)^∗^
42	Harpagoside	4.39	Terpenoids	494.17881	0.3	+Na	C_24_H_30_O_11_	Scrophulariae Radix	Positive	1.41766 (F6)^∗^
43	Benzoylpaeoniflorin	4.77	Terpenoids	584.18938	−1.1	+Na	C_30_H_32_O_12_	Moutan Cortex	Positive	2.03416 (F9)^∗^
44	Mudanpioside H	6.32	Terpenoids	616.17921	−4.3	+Na	C_30_H_32_O_14_	Moutan Cortex	Positive	
45	Galloylpaeoniflorin	6.33	Terpenoids	632.17412	2.4	+Na	C_30_H_32_O_15_	Moutan Cortex	Positive	
46	Tussilagolactone	6.49	Terpenoids	506.28797	−4.1	+NH4	C_28_H_42_O_8_	Farfarae Flos	Positive	1.47836 (F7)^∗^
47	Tussilagonone	9.41	Terpenoids	330.21949	−1	+NH4	C_21_H_30_O_3_	Farfarae Flos	Positive	0.492475 (F3)^∗^
48	α-Ionone	13.07	Terpenoids	192.15142	0.5	+Na	C_13_H_20_O	Canarii Fructus	Positive	0.257641 (F1)^∗^
49	1,2-Menthene	14.78	Terpenoids	152.12012	0.2	+NH4	C_10_H_16_O	Peppermint oil	Positive	
50	Ledol	14.79	Terpenoids	222.19837	0.3	+NH4	C_15_H_26_O	Canarii Fructus	Positive	
51	Geniposide	1.06	Terpenoids	449.1272	−2.9	+HCOO	C_17_H_24_O_10_	Stemonae Radix	Negative	
52	Harpagide	1.15	Terpenoids	363.1096	3.2	-H	C_15_H_24_O_10_	Scrophulariae Radix	Negative	
53	Mudanpioside G	1.19	Terpenoids	389.1439	−1.4	+HCOO	C_16_H_24_O_8_	Moutan Cortex	Negative	
54	Catalpol	1.56	Terpenoids	361.1206	−0.2	-H	C_15_H_22_O_10_	Rehmanniae Radix	Negative	
55	Deoxypaeonisuffrone	2.29	Terpenoids	181.0879	0.9	-H	C_10_H_14_O_3_	Moutan Cortex	Negative	0.690067 (F10)^∗^
56	Mudanpioside D	2.57	Terpenoids	555.1743	2.4	+HCOO, -H	C_24_H_30_O_12_	Moutan Cortex	Negative	
57	6-O-α-D-Galactopyranosyl harpagoside	6.59	Terpenoids	655.2264	−4.6	-H	C_30_H_40_O_16_	Scrophulariae Radix	Negative	
58	Caryophyllene oxide	7.1	Terpenoids	219.1772	1.7	-H,	C_15_H_24_O	Canarii Fructus	Negative	
59	Germacrene	10.34	Terpenoids	249.1846	−1.4	+HCOO	C_15_H_24_	Canarii Fructus	Negative	
60	Phenylpropionic acid_1	0.42	Organic acids	165.07898	0.2	+H	C_9_H_11_NO_2_	Peppermint oil, Cicadae Periostracum	Positive	1.35781 (F1)^∗^
61	Ferulic acid	0.47	Organic acids	194.05791	0.9	+NH4	C_10_H_10_O_4_	Scrophulariae Radix	Positive	0.518529 (F3)^∗^
62	Gallic acid	0.59	Organic acids	170.02152	−1.1	+H	C_7_H_6_O_5_	Canarii Fructus, Moutan Cortex	Positive	1.51267 (F2)^∗^
63	Chlorogenic acid	1.47	Organic acids	354.09508	0.4	+H	C_16_H_18_O_9_	Stemonae Radix	Positive	0.30626 (F4)^∗^
64	Rosmarinic acid	1.88	Organic acids	360.08452	−1.5	+H	C_18_H_16_O_8_	Peppermint oil	Positive	
65	Succinic acid	0.51	Organic acids	117.0204	1	-H	C_4_H_6_O_4_	Scrophulariae Radix, Farfarae Flos	Negative	
66	2-Methylbutyric acid	0.78	Organic acids	147.0662	0	+HCOO, -H	C_5_H_10_O_2_	Farfarae Flos	Negative	
67	3,5-O-Dimethyl gallic acid	0.83	Organic acids	243.0503	−0.7	+HCOO	C_9_H_10_O_5_	Isatidis Radix	Negative	
68	3-Methoxy-4-hydroxybenzoic acid	0.86	Organic acids	167.0349	−0.1	-H	C_8_H_8_O_4_	Scrophulariae Radix	Negative	0.867601 (F5)^∗^
69	3,4-Dihydroxycinnamic acid	1.69	Organic acids	179.0334	−1.6	-H	C_9_H_8_O_4_	Farfarae Flos	Negative	0.86764 (F6)^∗^
70	2-Hydroxy-1,4-benzene-dicarboxylic acid	1.9	Organic acids	181.0128	−1.5	-H	C_8_H_6_O_5_	Isatidis Radix	Negative	
71	Benzoic acid	2.26	Organic acids	121.0286	−0.9	-H	C_7_H_6_O_2_	Stemonae Radix, Oroxyli Semen, Isatidis Radix, Moutan Cortex	Negative	
72	*p*-Methoxycinnamic acid	2.68	Organic acids	223.0609	−0.3	+HCOO	C_10_H_10_O_3_	Scrophulariae Radix	Negative	
73	Menthyl benzoate	1.21	Volatile oils	136.05243	0.3	+H, +NH4	C_8_H_8_O_2_	Peppermint oil	Positive	
74	Benzyl alcohol	4.3	Volatile oils	108.05751	1.1	+Na	C_7_H_8_O	Farfarae Flos	Positive	
75	1-Octene	14.7	Volatile oils	112.1252	0.2	+H	C_8_H_16_	Farfarae Flos	Positive	
76	Stigmasterol	15.32	Volatile oils	412.37052	3.2	+H	C_29_H_48_O	Ophiopogonis Radix, Stemonae Radix	Positive	
77	Phenethyl alcohol	1.42	Volatile oils	167.0718	0.4	+HCOO	C_8_H_10_O	Farfarae Flos	Negative	
78	trans-Ocimene	5.25	Volatile oils	183.1381	−1	+HCOO	C_10_H_16_	Peppermint oil	Negative	
79	Menthyl acetate	6.35	Volatile oils	243.1596	−0.6	+HCOO	C_12_H_22_O_2_	Peppermint oil	Negative	
80	Methyl linoleate	6.8	Volatile oils	293.2514	2.8	-H	C_19_H_34_O_2_	Farfarae Flos	Negative	
81	Terpinyl acetate	7.08	Volatile oils	195.1392	0.1	-H	C_12_H_20_O_2_	Peppermint oil	Negative	
82	Methyl hexadecanoate	8.57	Volatile oils	315.2552	1.1	+HCOO	C_17_H_34_O_2_	Farfarae Flos	Negative	
83	6-Octadecenoic acid	13.52	Volatile oils	281.2485	−0.1	-H	C_18_H_34_O_2_	Canarii Fructus	Negative	
84	Tussilagine	0.41	Alkaloids	199.12084	−0.2	+H	C_10_H_17_NO_3_	Farfarae Flos	Positive	
85	Stemospironine	2.25	Alkaloids	351.20457	−2.3	+H	C_19_H_29_NO_5_	Stemonae Radix	Positive	
86	Tuberostemoenone	2.81	Alkaloids	387.20457	−2.9	+H	C_22_H_29_NO_5_	Stemonae Radix	Positive	
87	Oxotuberostemonine	2.95	Alkaloids	389.22022	−3.5	+H	C_22_H_31_NO_5_	Stemonae Radix	Positive	
88	N-Oxy-tuberostemonine	3.06	Alkaloids	391.23587	−3.4	+H	C_22_H_33_NO_5_	Stemonae Radix	Positive	
89	Tuberostemonone	5.52	Alkaloids	405.21514	−2.2	+H	C_22_H_31_NO_6_	Stemonae Radix	Positive	
90	Senkirkine	2.79	Alkaloids	366.0904	−0.7	-H	C_19_H_28_NO_6_	Farfarae Flos	Negative	
91	Oxymaistemonine	4.29	Alkaloids	430.193	−0.9	-H	C_23_H_29_NO_7_	Stemonae Radix	Negative	
92	14-Hydroxy sprengerinin C	4.36	Saponins	870.4613	−8	+H	C_44_H_70_O_17_	Ophiopogonis Radix	Positive	
93	Ophiopogonin C′	5.4	Saponins	722.42413	−4.8	+H	C_39_H_62_O_12_	Ophiopogonis Radix	Positive	
94	Ophiopogonin B	6.03	Saponins	722.42413	−4.9	+H	C_39_H_62_O_12_	Ophiopogonis Radix	Positive	
95	Ophiopogonin A	6.39	Saponins	764.43469	−3.7	+NH4	C_41_H_64_O_13_	Ophiopogonis Radix	Positive	
96	Ophiopogonin E	6.55	Saponins	724.40339	4.6	+NH4	C_38_H_60_O_13_	Ophiopogonis Radix	Positive	
97	Ophiopogonin D	8.38	Saponins	899.471	3.4	+HCOO	C_44_H_70_O_16_	Ophiopogonis Radix	Negative	
98	Sprengerinin C	8.42	Saponins	899.4718	4.2	+HCOO	C_44_H_70_O_16_	Ophiopogonis Radix	Negative	
99	Smilagenin	13.88	Saponins	415.3207	−1.1	-H	C_27_H_44_O_3_	Asparagi Radix	Negative	
100	Adenosine	0.46	Glycosides	267.09675	−1.9	+H	C_10_H_13_N_5_O_4_	Isatidis Radix	Positive	
101	n-Butanol-β-D-fructopyranoside	0.62	Glycosides	236.12599	0.5	+NH4	C_10_H_20_O_6_	Peppermint oil	Positive	
102	Ningpogenin	1.52	Glycosides	170.09429	1.7	+Na	C_9_H_14_O_3_	Scrophulariae Radix	Positive	
103	Rehmannioside D	6.56	Glycosides	686.22694	0	+H, +Na	C_27_H_42_O_20_	Rehmanniae Radix	Positive	
104	Verbascoside	5.85	Glycosides	623.1048	−0.1	-H	C_29_H_36_O_15_	Scrophulariae Radix, Rehmanniae Radix	Negative	
105	Mudanoside A	0.32	Acetophenones	330.09508	1	+Na	C_14_H_18_O_9_	Moutan Cortex	Positive	
106	Sucrose	0.34	Saccharides	342.11621	0.3	+Na	C_12_H_22_O_11_	Moutan Cortex	Positive	
107	Vitamin B5	0.37	Vitamins	219.11067	0.1	+Na, +H	C_9_H_17_NO_5_	Canarii Fructus	Positive	
108	3,3′-Di-O-methylellagic acid	0.39	Tannins	330.03757	−3	+NH4	C_16_H_10_O_8_	Canarii Fructus	Positive	
109	Scoparone	0.45	Phenylpropanoids	206.05791	0.5	+NH4	C_11_H_10_O_4_	Canarii Fructus	Positive	
110	Apiopaeonoside	1.91	Acetophenones	460.15808	−2.8	+Na	C_20_H_28_O_12_	Moutan Cortex	Positive	
111	Ellagic acid	2.46	Tannins	302.00627	−0.2	+H	C_14_H_6_O_8_	Canarii Fructus	Positive	
112	1,2,3,4,6-Penta-O-galloyl-β-D-glucopyranoside	6.44	Tannins	940.11818	4	+H	C_41_H_32_O_26_	Moutan Cortex	Positive	
113	Verbascose	6.47	Saccharides	828.27468	4.3	+Na	C_30_H_52_O_26_	Rehmanniae Radix	Positive	
114	Caproic acid	6.51	Fatty acids	116.08373	0.3	+H	C_6_H_12_O_2_	Canarii Fructus	Positive	
115	Hexadecanoic acid	7.02	Fatty acids	256.24023	0.7	+NH4	C_16_H_32_O_2_	Canarii Fructus, Asparagi Radix	Positive	
116	Octadeca-carboxylic acid	8.44	Fatty acids	284.27153	−2.8	+NH4	C_18_H_36_O_2_	Canarii Fructus	Positive	
117	Stilbostemin B	10.28	Stilbenes	228.11503	0.1	+H	C_15_H_16_O_2_	Stemonae Radix	Positive	
118	Ursolic acid	14.59	Triterpenoids	456.36035	−1.8	+Na	C_30_H_48_O_3_	Peppermint oil, Scrophulariae Radix	Positive	
119	Urs-12-ene-3β,16β-diol	15.28	Triterpenoids	442.38108	1.8	+Na	C_30_H_50_O_2_	Canarii Fructus	Positive	
120	Arnidiol	15.52	Triterpenoids	442.38108	3.7	+Na	C_30_H_50_O_2_	Farfarae Flos	Positive	
121	Proline	0.33	Amino acids	160.0613	−0.3	+HCOO	C_5_H_9_NO_2_	Peppermint oil, Cicadae Periostracum	Negative	
122	Arabinose	0.35	Saccharides	195.0503	−0.8	+HCOO, -H	C_5_HO_5_	Moutan Cortex	Negative	
123	Tryptanthrin	0.53	Indoles	247.0494	−1.9	-H	C_15_H_8_N_2_O_2_	Isatidis Radix	Negative	
124	Norleucine	0.54	Amino acids	130.0877	0.3	-H	C_6_H_13_NO_2_	Cicadae Periostracum	Negative	
125	5-Hydroxymethyl furoic acid	0.81	Furans	141.0202	0.9	-H	C_6_H_6_O_4_	Isatidis Radix	Negative	
126	Cystine_1	1.66	Amino acids	239.0179	1.3	-H	C_3_H_7_NO_2_S	Peppermint oil, Cicadae Periostracum	Negative	
127	Physcion-8-O-β-D-glucoside	2.58	Quinones	431.0974	−1	-H	C_22_H_22_O_10_	Isatidis Radix	Negative	
128	Nicotinic acid	3.85	Vitamins	168.0304	0.1	+HCOO	C_6_H_5_NO_2_	Isatidis Radix	Negative	
129	Paeonol	4.37	Acetophenones	165.0543	−1.5	-H	C_9_H_10_O_5_	Moutan Cortex	Negative	
130	Emodin	4.62	Quinones	269.0463	0.7	-H	C_15_H_10_O_5_	Peppermint oil, Isatidis Radix	Negative	
131	Dibutyl phthalate	8.73	Organic esters	277.143	−1.5	-H	C_16_H_22_O_4_	Farfarae Flos	Negative	

“⁣^∗^” indicates that the ingredient is common to the samples of each enterprise.

## Data Availability

The data used to support the findings of this study are included in the article. Any further information is available from authors upon request.

## Nomenclature

VIP: Variable importance in projection
OPLS–DA: Orthogonal partial least squares discriminant analysis
HPLC: High-performance liquid chromatography
HPLC–DAD: High-performance liquid chromatography with diode array detector
PCA: Principal component analysis
QC: Quality control
NIFDC: National Institutes for Food and Drug Control
UPLC: Ultra-performance liquid chromatography
ESI: Electrospray ionization
UV: Ultraviolet
RSDs: Relative standard deviations
HCA: Hierarchical cluster analysis
TCM: Traditional Chinese medicine
IDL: Instrument detection limit
MDL: Method detection limit
UPLC–Q–TOF–MS/MS: Ultra-performance liquid chromatography–quadrupole time-of-flight tandem mass spectrometry
